# Case of Vertebral Dislocation

**Published:** 1855-04

**Authors:** 


*Case of Vertebral Dislocation.*—Dr. J. M. Hickerman, of Tiffin City, Ohio, reports to the Ohio Journal a case of this formidable accident. The skin was cool, pupils dilated, heart's motion barely perceptible, and respiration occurred only at long intervals. Dr. H. found, by exploring the pharynx, a prominence opposite the junction of the fourth and fifth cervical vertebra:

"After a little reflection I seized the patient's head under my left arm, with which I made traction, and with the index finger of the right hand in the patient's throat, I made firm pressure obliquely upward, backward and to the left; after continuing this pressure for about forty or fifty seconds, the part against which the finger was placed gradually, yet quickly, receded in the direction in which the pressure was made, and instantly, as quickly indeed as the act could possibly be executed, the patient opened her eyes, and natural respiration was established. The patient immediately became conscious of what was transpiring about her, recognized the persons present, and upon being asked if she was suffering any pain, signified by signs (being unable to speak,) that she had, and when asked its locality, referred to the epigastric and hypochondriac regions."

Complete recovery took place. H.

---

We have received number one of volume one of "The Family Guide to Health and Happiness.

'Health is the Charm of Life.'

'Why shouldst thou die before thy time ?'—Jehovah.*

'We labor to promote that good time coming, foretold by the prophet, when a man at one hundred years shall be called an infant, and when the 'age of the people shall be as the age of a tree.' —Editor.*

Rev. Samuel Kyle, M. D., Editor."

Not a month passes without our receiving a favor of this kind from the eclectic gentry; and over and over again, as they perish from the earth, dying from lack of sustenance in the shape of subscribers, we have asked the question, "why shouldst thou die before thy time?"

This new aspirant for eclectic honors is devoted to longevity. If it does not furnish in itself a better exemplification of long life than most—we may

---

* What a blasphemous association of authorities!—Printer's Devil.

say all—of its fellow-laborers in the cause of skunk's-cabbage and mandrake, it will struggle along for a brief season, become irregular and intermittent, reviving occasionally to assure its readers that temporary and unforeseen obstacles have been overcome, and that it is "finally established on a basis of the firmest footing," and then, in hospital slang, it will "peg," and disappear forever. H.

---

The Boston Medical Journal of March 8, has a masterly review of Dr. Holmes' "Puerperal Fever as a Private Pestilence." The new accessions to its editorial corps seem likely to revolutionize its pages. The valuable reports of the Transactions of the "Society for Medical Improvement" are no longer sent to Philadelphia for publication, and it is easy to recognize in the tone of the Journal a new and progressive spirit. *Perge et valete!*

Another recent number of the same Journal has a review of Dr. Barton's Sanitary Report, or rather a review of an ill-tempered and unjustifiable criticism upon it, in the New Orleans Journal. Our opinions of the high value of Dr. Barton's report have been so freely expressed that it is unnecessary to repeat them, and we only allude to the matter, that we may gain the opportunity to endorse the spirit and matter of the Bostonian reviewer. H.

